# Plant cell wall integrity maintenance in model plants and crop species-relevant cell wall components and underlying guiding principles

**DOI:** 10.1007/s00018-019-03388-8

**Published:** 2019-11-28

**Authors:** Nora Gigli-Bisceglia, Timo Engelsdorf, Thorsten Hamann

**Affiliations:** 1 Laboratory of Plant Physiology, Wageningen University and Research, Wageningen, 6708 PB The Netherlands; 2 Division of Plant Physiology, Department of Biology, Philipps University of Marburg, 35043 Marburg, Germany; 3 Institute for Biology, Faculty of Natural Sciences, Norwegian University of Science and Technology, 5 Høgskoleringen, 7491 Trondheim, Norway

**Keywords:** Plant cell wall metabolism, Plant cell wall signaling, Cell wall polysaccharides, Plant defense, Plant environment interaction

## Abstract

The walls surrounding the cells of all land-based plants provide mechanical support essential for growth and development as well as protection from adverse environmental conditions like biotic and abiotic stress. Composition and structure of plant cell walls can differ markedly between cell types, developmental stages and species. This implies that wall composition and structure are actively modified during biological processes and in response to specific functional requirements. Despite extensive research in the area, our understanding of the regulatory processes controlling active and adaptive modifications of cell wall composition and structure is still limited. One of these regulatory processes is the cell wall integrity maintenance mechanism, which monitors and maintains the functional integrity of the plant cell wall during development and interaction with environment. It is an important element in plant pathogen interaction and cell wall plasticity, which seems at least partially responsible for the limited success that targeted manipulation of cell wall metabolism has achieved so far. Here, we provide an overview of the cell wall polysaccharides forming the bulk of plant cell walls in both monocotyledonous and dicotyledonous plants and the effects their impairment can have. We summarize our current knowledge regarding the cell wall integrity maintenance mechanism and discuss that it could be responsible for several of the mutant phenotypes observed.

## Introduction

Plants represent essential sources of food and produce feedstocks for biofuel and fine chemical production [1]. To improve food crop performance and facilitate bioenergy production, significant efforts have been made to modify plant cell wall composition. However, these efforts have yielded mixed results [2, 3]. The reason seems to be that plant cell walls are extremely plastic and effects of the genetically induced changes are limited by the modification of other cell wall components. This seems to be comparable to processes taking place during development and interaction with the environment, where perception of physical and chemical stimuli leads to highly adaptive and tightly controlled changes in cell wall composition and structure. The tight control is essential because turgor pressure levels in plant cells are often equivalent to pressure levels in car tires, meaning that uncontrolled/accidental weakening of cell walls leads to cell bursting and cell death. Currently it seems that the plant cell wall integrity (CWI) maintenance mechanism forms an essential component of cell wall plasticity by being responsible for perceiving stimuli indicating CWI impairment and initiating adaptive responses in cellular and cell wall metabolism leading to changes in wall composition and structure.

Different polysaccharides and proteins form the primary and secondary cell walls surrounding land plant cells. Primary cell walls of *Arabidopsis thaliana* (Arabidopsis, serving here as representative example for dicotyledonous plants) contain mostly pectins [rhamnogalacturonan-I (RG-I 11%), rhamnogalacturonan-II (RG-II 8%), and homogalacturonan (HG 23%)] followed by 24% hemicellulose and 14% cellulose [4]. Primary cell walls of grasses, here representing monocotyledonous plant species, contain mostly hemicellulose (20–45%; including different types of xylans), cellulose (20–30%), pectins (5–10%) and in *Poaceae* and related families, mixed-linkage glucans (MLGs), which are absent in dicots [5, 6]. Secondary cell walls are deposited once cells have terminally differentiated and consist in Arabidopsis mainly of cellulose (40–80%), lignin (5–25%) and hemicellulose (10–40%) [7]. In addition, cell wall-specific proteins like extensins, expansins, hydroxyproline- and glycine-rich proteins and dynamically formed polysaccharides like callose can be also found in cell walls and will not be covered here in detail since they have already been recently reviewed [7, 8]. This simplified global overview summarizes the main components, which form the bulk of the primary and secondary cell walls in plants and could therefore be relevant in the context of CWI maintenance.

Here, we will initially review processes giving rise to the main cell wall components and assess the consequences their impairment has on plant growth, development and stress responses. Since knowledge about primary cell walls is most extensive in Arabidopsis, we will use them as baseline for comparison purposes with knowledge on cell walls in other plant species (both mono- and dicots). This will also enable us to provide perspective about components possibly involved in or affected by CWI maintenance. We will finish by summarizing the current knowledge about the CWI maintenance mechanism and discuss concepts for its mode of action.

### Cellulose biosynthesis in dicots

**Fig. 1:**
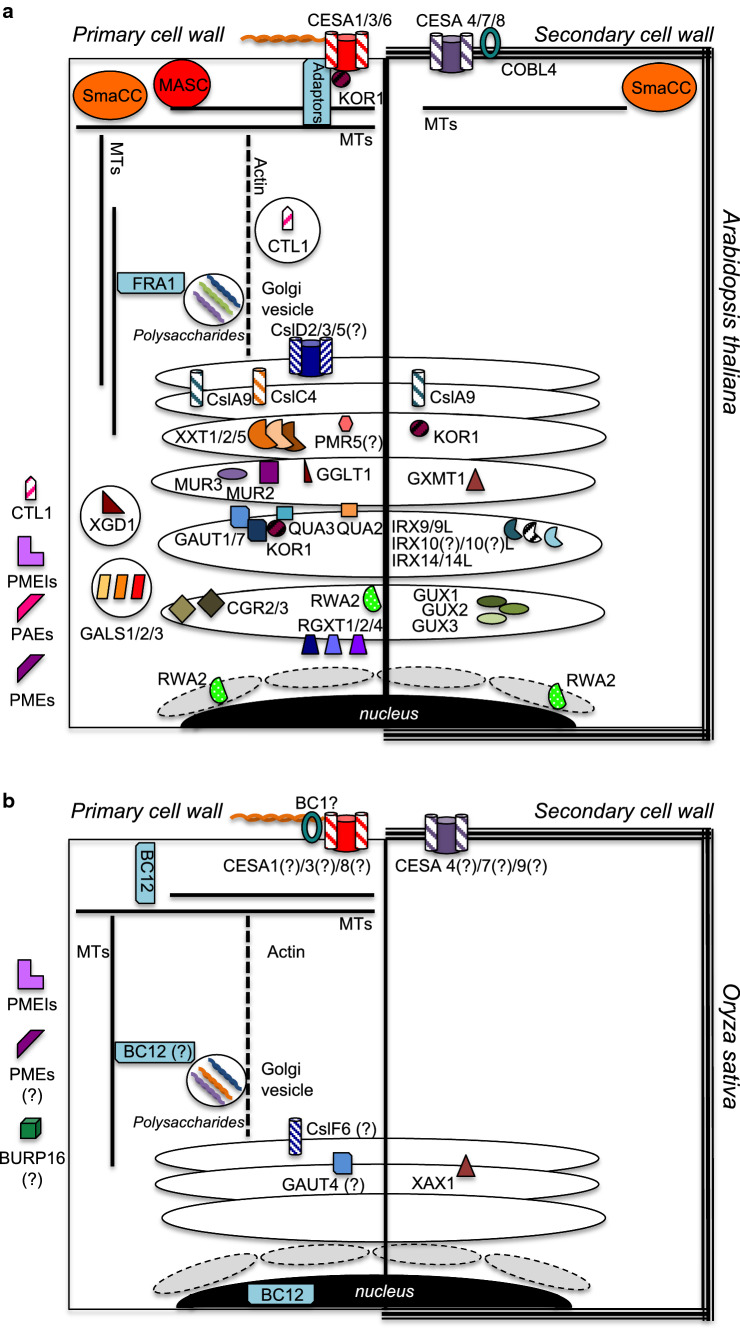
Global overview of proteins mediating primary (PCW) and secondary cell wall (SCW) formation in dicots (Arabidopsis) and monocots (Oryza). Demonstrated or putative (indicated by ?) protein localizations are displayed in panel **a** based on data mostly derived from Arabidopsis. Enzymes such as PMEIs, PAEs and PMEs, which have been found in the apoplast, are listed outside the cells in panels **a** and **b**. Ellipses represent Golgi bodies/stacks with enzymes located to highlight their positions either inside the Golgi or associated with Golgi membranes. Circles containing enzymes like GALS1/2/3 or CTL1 represent Golgi-derived vesicles. Grey, dashed circles near the nucleus (black ellipse) represent the endoplasmic reticulum. **b** Summarizes putative localizations of proteins in Oryza. Abbreviations are explained in main text. Protein localizations are based on the following references: [16, 34, 45, 47, 68, 74, 104, 125, 138, 155, 156, 160, 161, 163, 164, 167, 168, 171, 180, 255–270]

The primary cell wall in Arabidopsis consists of a framework of cellulose microfibrils cross-linked by xyloglucans and embedded in a matrix of acid-rich pectic polysaccharides [8, 9]. Primary cell walls are produced right after cell division and during cell elongation, highlighting the involvement of the walls in cell morphogenesis and the need for extensibility. Cellulose is the most abundant water-insoluble polymer found in nature. This linear polymer consists of β (1 → 4) linked d-glucose units and is synthesized by the plasma membrane-localized cellulose synthase complexes (CSCs). CSCs are transmembrane structures consisting of several CELLULOSE SYNTHASE A (CESA) proteins organized in a rosette shape associated with a large number of other proteins [10–12] (Fig. 1; Table 1). Ten CESA proteins have been identified in Arabidopsis. AtCESA1, AtCESA3 and AtCESA6 are involved in cellulose synthesis during primary cell wall, while AtCESA4, AtCESA7 and AtCESA8 are active during secondary cell wall establishment [13–15]. AtCESA6 can be replaced to some extent by AtCESA2, AtCESA5 and AtCESA9, suggesting partially redundant roles in primary cell wall CSCs [16, 17], while the biological role of AtCESA10 remains unclear [18]. Detailed structural analyses showed that all AtCESAs have eight transmembrane domains (TMDs), with two being located near the N-terminal region and six near the C-terminus [19]. Between TMD2 and TMD3 resides a large, highly conserved cytosolic region, responsible for uridine diphosphate (UDP) glucose binding and catalysis. By now a large number of mutations in At*CESA* genes have been isolated, providing insights into the importance of the different domains within the CESA proteins through the mutant phenotypes caused (ranging from very mild to radial cell swelling and stunted growth) [20]. While knockout (KO) alleles for At*CESA1* and At*CESA3* lead to lethality, plants with At*CESA6* KO alleles are viable and exhibit only limited cell elongation defects (i.e., At*CESA6*^*prc1*−*1*^ to At*CESA6*^*prc1*−*12*^) [17, 20]. More recently, cellulose biosynthesis inhibitors (CBIs) have been combined with genetic approaches to analyze CESA activity as well as the responses to cell wall damage (CWD) induced by cellulose biosynthesis inhibition [21]. This is exemplified by isoxaben (ISX, a CBI), which was used to isolate At*CESA3*^*ixr1*−*1*^, characterized by an amino acid substitution at Glycine-998 located at the start of TMD8, causing resistance to ISX. The mutation leads also to reduced levels of relative cellulose crystallinity but does not cause major growth defects [22, 23]. Impairing cellulose biosynthesis induces a variety of responses in plants. This is illustrated by the At*CESA1*^*any1*^, At*CESA6*^*eli1*^ and At*CESA6*^*cev1*^ mutations [24–26]. At*CESA1*^*any1*^ was originally implicated in microtubule organization in epidermal cells. At*CESA6*^*eli1*^ was implicated in cell morphogenesis because of ectopic lignin deposition, whereas At*CESA6*^*cev1*^ seemed required for pathogen response since it causes constitutive expression of *VEGETATIVE STORAGE PROTEIN1* (*VSP1,* implicated in pathogen defense) and production of jasmonic acid (JA) [24, 26]. ISX, which inhibits cellulose biosynthesis in primary cell walls, triggers responses similar to the ones induced by the mutations described. Responses include induction of defense gene expression, phytohormone accumulation such as salicylic acid (SA)/JA (as observed in At*CESA6*^*cev1*^) and lignin accumulation (as in At*CESA6*^*eli1*^) [24, 26–29]. Observing these phenotypes led to the suggestion that a CWI maintenance mechanism, similar to the one described in yeast, may exist in plants [30]. This mechanism involves constant monitoring of the functional integrity of the cell wall and triggering of apparently compensatory responses to maintain integrity during development and interaction with the environment [28].

**Table 1:** List of the genes and mutant phenotypes described

Name	Mutant	Mutant phenotype	Reference
AtCESA1	*rsw1, any1, fxr2*	Reduced cellulose content in primary cell walls, reduced growth, radial cell swelling (*rsw1)* Reduced growth, swollen roots, bulging epidermal cells in cotyledons and reduced crystalline cellulose content (*any1*) Resistance to *flupoxam,* smaller rosettes and reduced crystalline cellulose (*fxr2*). CESA1 null mutants are lethal	[22, 271, 272]
AtCESA2	*cesa2*	Phenotypically normal. *CESA2* overexpression in *cesa6* null mutants can rescue to some extent the *cesa6*-dependent growth defects	[20]
AtCESA3	*cev1, eli1, ixr1*	Seedlings exhibit constitutive JA and ET accumulation, reductions in cellulose levels, are smaller and have roots thicker than wt (*cev1*) Reduced plant growth, ectopic lignification and reduced cellulose content in seedlings (*eli1*) *ixr1* plants are phenotypically normal, exhibit slight reductions in crystalline cellulose levels and resistance to ISX	[22–24, 26]
AtCESA4	*irx5*	Reduced cellulose levels in secondary cell walls. Defects in cell wall thickness in xylem vessels. Reduced mechanical strength of the stems. Adult plants are smaller than wt	[273]
AtCESA5	*cesa5*	Mutant plants exhibit defective seed coat mucilage synthesis. *CESA5* overexpression in *cesa6* null mutants can rescue partially *cesa6*-dependent growth defects	[20]
AtCESA6	*prc1, ixr2*	Defects in cellulose production during primary cell wall formation, short hypocotyls and ectopic lignification (*prc1*) No obvious growth phenotypes but ISX resistant (*ixr2*)	[23, 274, 275]
AtCESA7	*irx3*	Reduced cellulose levels in secondary cell walls. Defects in cell wall thickness in xylem vessels. Reduced mechanical strength of the stems. Adult plants are smaller than wt	[13]
AtCESA8	*irx1*	Reduced cellulose in secondary cell walls. Defects in cell wall thickness in xylem vessels. Reduced mechanical strength of the stems. Adult plants are smaller than wt	[15]
AtCESA9	*cesa9*	Mutant plants display wt-like phenotype. Epidermal cells in seed coats of *cesa9* seeds are distorted	[276]
AtCESA10	*cesa10*	No obvious mutant phenotypes	[17, 18]
KORRIGAN	*kor1*	Dwarf, cellulose deficient, display defects during cytokinesis	[47, 277]
KOBITO	*kob1*	Dwarf, sterile, short hypocotyls, incomplete cell wall during cytokinesis, cellulose deficient	[48]
CHITINASE-LIKE 1	*ctl1/pom*-*pom1*	Cellulose deficient, the mutation affects xyloglucan structure. Mutant plants are smaller than wt, have shorter hypocotyls and swollen roots. *ctl1* phenotype is rescued by overexpressing *CTL2*	[49, 52]
CHITINASE-LIKE 2	*ctl2*	Mutant plants are similar to the wt but display ectopic lignification in stems and etiolated hypocotyls	[49]
CELLULOSE SYNTHASE INTERACTING1	*csi1/pom*-*pom2*	Dwarf, short hypocotyls, swollen cells, altered microtubule organization and cellulose deficient	[52, 278]
MUNC13-LIKE PROTEIN	*patrol1*	Cellulose deficient, mutants are smaller than wt. This mutant displays short hypocotyls and roots. Adult plants are significantly smaller than the wt	[32, 279]
STELLO1	*stl1*	No obvious mutant growth phenotype. Functionally redundant with STL2	[34]
STELLO2	*stl2*	No obvious mutant growth phenotype. Functionally redundant with STL1, double *stl1stl2* mutants are small, display cellulose deficiency and reduced cell wall thickness	[34]
KINESIN-LIKE PROTEIN	*fra1*	Dwarf plants with reduced mechanical strength. Mutant plants display changes in cellulose orientation with no overall reduction of cellulose	[45]
CELLULOSE SYNTHASE-LIKE PROTEIN D2	*csld2*	Root hair formation defective with altered cytoskeleton organization	[258]
CELLULOSE SYNTHASE-LIKE PROTEIN D3	*csld3/kjk*	Root hair formation defective resulting in root hair rupture	[74, 258]
CELLULOSE SYNTHASE-LIKE PROTEIN D5	*csld5*	No obvious growth phenotype, required for cell plate formation	[75, 280]
CELLULOSE SYNTHASE-LIKE PROTEIN A7	*csla7*	Pollen tube growth defects and embryogenesis failure. Overexpression of *CSLA9* rescues *csla7* mutant phenotype	[80]
CELLULOSE SYNTHASE-LIKE PROTEIN A9	*csla9*	Reduced glucomannan. No obvious mutant phenotypes	[79, 80]
XYG-XYLOSYLTRANSFERASE	*xxt1*	No obvious mutant phenotypes	[105]
XYG-XYLOSYLTRANSFERASE	*xxt2*	No obvious mutant growth phenotypes. Double *xxt1 xxt2* show smaller rosettes and collapsed stems. Seedling hypocotyls are smaller and thicker than the wt. Cellulose deficiency associated with cellulose fibril and cortical microtubules disorganization. Lack detectable xyloglucan	[105, 281]
XYLOGLUCAN GALACTOSYLTRANSFERASE	*mur3*-*3*	Cabbage-like phenotype, smaller plants with smaller rosettes. Altered xyloglucan composition. MUR3-dependent phenotype can be rescued by knocking out *XXT2* alone or together with *XXT1*	[107, 111]
FUCOSYLTRANSFERASE 1	*mur2*	No obvious mutant growth phenotype, impaired in xyloglucan fucosylation	[172, 282, 283]
PUTATIVE FAMILY 43 GLYCOSYL TRANSFERASE	*irx9*	Irregular xylem phenotype, reduced cell wall thickness due to xylan chain elongation defects. Reduced xylose levels	[267]
PUTATIVE FAMILY 43 GLYCOSYL TRANSFERASE	*irx9*-*like*	No obvious mutant phenotypes	[124]
PUTATIVE FAMILY 43 GLYCOSYL TRANSFERASE	*irx14*	Slightly smaller plants, with thinner vessels. Reduced xylose levels	[123, 124]
PUTATIVE FAMILY 43 GLYCOSYL TRANSFERASE	*irx14*-*like*	No obvious mutant phenotypes	[123]
PUTATIVE FAMILY 47 GLYCOSYL TRANSFERASE	*irx10*	Overall normal plants with reduced xylose levels associated with a mild irregular xylem phenotype	[127]
PUTATIVE FAMILY 47 GLYCOSYL TRANSFERASE	*irx10*-*like*	No obvious mutant phenotypes	[127]
GLUCURONYL TRANSFERASE	*gux1*	No obvious mutant growth phenotype. Significantly reduced levels of GlcA-substituted xylan	[284]
GLUCURONYL TRANSFERASE	*gux2*	No obvious mutant phenotype. Significantly reduced levels of GlcA-substituted xylan. Double *gux1/gux2* mutants lack almost all detectable xylan substitutions	[125, 284]
GLUCURONYLTRANSFERASE	*gux3*	No obvious mutant growth phenotype. Significantly reduced levels of GlcA-substituted xylan. Triple *gux1gux2 gux3* mutants are small and have reduced resistance to breaking force	[133]
GLUCURONOXYLAN-METHYLTRANSERASE	*gxmt1*-*1*	No obvious mutant growth phenotype. Reduced methylation of glucuronoxylan	[138]
GALACTURONOSYLTRANSFERASE 1-GAUT 8	*qua1/gaut8*	Significantly reduced growth and decrease in both HG and xylan-synthase activity	[159]
GALACTURONOSYLTRANSFERASE 10-GAUT 10	*gaut10*	Mutant seedlings exhibit growth defects and short roots in the absence of sucrose	[162]
GALACTURONOSYLTRANSFERASE 11-GAUT 11	*gaut11*	Identified as HG α-GalA transferase. KO mutant seeds produce less RG I and mucilage in coat epidermal cells (SCE)	[167]
PUTATIVE GLYCOSYLTRANSFERASE	*muci70*	Required for pectin synthesis in seed coat epidermal cells. KO mutant seeds produce less mucilage	[167]
PUTATIVE METHYLTRANSFERASE	*qua2*	Defective in cell adhesion, characterized by reduced HG content	[285]
PUTATIVE HOMOGALACTURONAN-METHYLTRANSFERASE	*qua3*	No obvious growth defects and normal pectin methylation in KO plants. *QUA3* RNAi suspension cells exhibit reduced pectin methylation	[156]
PECTIN METHYLTRANSFERASE-CGR2	*cgr2*	No obvious mutant growth phenotype. Slight reduction in uronic acids and pectin methylation	[161]
PECTIN METHYLTRANSFERASE- CGR3	*cgr3*	No obvious growth phenotypes. *cgr2 cgr3* plants are dwarf, display reduced cell elongation. Double mutant plants show significant reduced degree of methylesterification, and uronic acids content compared to the single mutants, in addition to a slight cellulose reduction	[161]
XYLOGALACTURONAN-XYLOSYLTRANSFERASE	*xgd1*	No obvious mutant growth phenotype. Reduced xylose levels associated with a reduction in xylogalacturonan content	[163]
GOLGI GDP-l-GALACTOSE TRANSPORTER1	*gglt1/gonst3*	Mutant growth phenotype is rescued by application of boric acid. Mutant lines exhibit reduced levels of L-galactose in side-chain A of RG-II	[171]
GDP-MANNOSE 4,6 DEHYDRATASE ACTIVITY	*mur1*	KO plants are defective in L-fucose, content in both pectin and hemicellulose. Reduced strength to mechanical force. Phenotype can be rescued by boric acid application	[171, 173]
GALACTAN SYNTHASE 1	*gals1*	No obvious mutant growth phenotype. KO plants display decreased β-1,4-galactan content. Reduced galactose in stems. Decreased galactose in seeds	[164]
GALACTAN SYNTHASE 2	*gals2*	No obvious mutant growth phenotype but decreased β-1,4-galactan content. Decreased galactose levels in seeds	[164]
GALACTAN SYNTHASE 3	*gals3*	No obvious mutant growth phenotype. Mutant plants display decreased β-1,4-galactan content. Reduced galactose in stems	[164]
RHAMNOGALACTURONAN-II XYLOSYLTRANSFERASE-1	*rgxt1*	No obvious mutant growth phenotype. Involved in the xylosylation of the internal fucose moiety RG II	[168]
RHAMNOGALACTURONAN-II XYLOSYLTRANSFERASE-2	*rgxt2*	No obvious mutant growth phenotype. Involved in the xylosylation of the internal fucose moiety RG II	[168]
RHAMNOGALACTURONAN-II XYLOSYLTRANSFERASE-3	*rgxt4/mgp4*	Short siliques. KO plants are impaired in pollen tube growth	[170]
*O*-ACETYLTRANSFERASE	*rwa2*	No obvious mutant growth phenotype. KO plants characterized by reduced wall acetylation of both pectic and non-pectic polysaccharides. KO plants show increase resistance to *B.cinerea*	[142]
*O*-ACETYLTRANSFERASE	*tbl10*	No obvious mutant growth phenotype. KO plants show reduced RG I acetylation and enhanced drought stress resistance	[175]
*O*-ACETYLTRANSFERASE	*tbl44/pmr5*	KO plants show reduced rosette size. Enhanced resistance to powdery mildew and *C. higginsianum.* KO plants exhibit reduced pectin methyl esterification/O-acetylation associated with increased amount of pectins	[151, 177]
MURUS8	*mur8*	KO plants have no obvious mutant growth phenotype but exhibit reductions in rhamnogalacturonan-I and rhamnose content as well as enhanced resistance to *C. higginsianum*	[151, 179, 282]
PECTIN METHYLESTERASE 5	*pme5*	Involved in regulating HG methylesterification in stems. *PME5* expression is reduced in the internodes of *blr*-*6* mutants leading to reduced cell expansion and internode elongation	[286]
PECTIN METHYLESTERASE INHIBITOR 3	*pmei3*	Involved in regulating pectin de-metylesterification in apical meristems. *PMEI3* overexpression inhibits primordial formation	[287]
PECTIN METHYLESTERASE 35	*pme35*	KO plants exhibit reduced mechanical strength in the basal part of the inflorescence stems. Reduced cell wall thickness in cortical cells of mature stems, possibly due to changes HG de-methylesterification	[190]
RECEPTOR-LIKE PROTEIN 44	*rlp44/cnu2*	Adult KO plants exhibit stunted growth, reduced petiole length and rosette diameter. Mutant seedlings show reduced root and hypocotyl length in high sucrose levels as well as salt oversensitivity	[192]
WALL-ASSOCIATED KINASE 1	*wak1*	No obvious mutant growth phenotype. WAK1 overexpression leads to enhance callose deposition in response to wounding and exogenous OG application. WAK1 overexpression is associated with enhanced *B. cinerea* resistance	[288]
WALL-ASSOCIATED KINASE 2	*wak2*	In the absence of sucrose *wak2* KO seedlings are characterized by stunted growth and short roots. Exogenous application of metabolic active sugars (fructose or glucose) helps in rescuing *WAK2*-dependent cell elongation defects	[152]
OsCESA7 (rice)	*S1*-*24*	Smaller rice plants with brittle culm phenotype. Reduced resistance to mechanical force and cellulose deficient	[63]
OsCESA4 (rice)	*brittle culm7/bc7(t)*	Cellulose reduction and overall fragility of the mutant plants. Cellulose reduction is associated to enhanced lignin accumulation in stems and leaves	[289]
COBRA-like protein (rice)	*brittle culm1*	Cellulose deficient enhanced lignification and reduced resistance to mechanical force associated to brittle culm phenotype. Exhibit reduced leaf elongation	[66]
COBRA-like protein—(rice)	*brittle culm5*	Brittle culm phenotype, reduced lignification of the nodes. Cellulose and glucuronosyl arabinoxylan deficient	[64]
GA20-oxidase (Sorghum)	*dwf1*-*1*	Cellulose deficient mutant, dramatically reduced growth, gibberellin synthesis impaired. This mutation affects the expression of Sorghum *CESA1, 3* and *6*	[290]
Cellulose synthase-like F6 (rice)	*cslF6*	Reduced plant height and smaller stems. Impaired in MLG synthesis	[89]
KINESIN-LIKE PROTEIN (rice)	*brittle culm 12*	Dramatically reduced growth, with short roots and internodes. Culms display brittle phenotype with reduced cell wall strength	[68]
GLYCOSYLTRANSFERASE FAMILY 61(rice)	*xax1*	Mutant plants exhibit reduced growth. Xylose-deficient loss-of-function rice mutant. *xax1* plants are deficient in ferulic and coumaric acid	[269]
PECTIN ACETYLESTERASE1 (black cottonwood)	PtPAE1	Overexpression in tobacco leads to reduction in acetyl groups of pectins	[182]
GALACTURONOSYLTRANSFERASE 4 (switchgrass, poplar)	*KD*-*GAUT4 (RNAi) gaut4*	Involved in the synthesis of HG. Reduced expression of *GAUT4* in switchgrass and poplar associated with reduced HG and RG II content. KD transgenic plants show enhanced growth when compared to wt plants	[202]
POLYGALACTURONASE 1 *β* SUBUNIT	*OE:OsBURP16*	Rice plants overexpressing BURP16 are phenotypically normal. At cellular level *oeOsBURP16* transgenic plants show reduced cell adhesion and reduced resistance to breaking force	[207]

Loss of cellulose integrity affects also microtubule dynamics [31]. While it is not clear how ISX or genetic inhibition of cellulose synthesis affects microtubule orientation, the importance of the cytoskeleton in delivering CSCs to/recycling them at the PM has been reported [32–34] (Fig. 1). Golgi-derived vesicles containing CSCs interact with cortical microtubules through adaptors/docking sites, involving several proteins (CELLULOSE SYNTHASE INTERACTIVE 1 (CSI1/POM2), COMPANION OF CELLULOSE SYNTHASE (CC), PATROL1, STELLO1/2 and the EXOCYST COMPLEX) [32, 34–36]. These components control CSC delivery rate and allow CSC-containing vesicles to fuse with the plasma membrane. Mechanical distortion of cell walls and/or loss of CWI (i.e., osmotic stress or CBI treatment) trigger CESA re-localization to intracellular compartments [37, 38]. These have been termed small CESA containing compartments (SmaCCs) and when associated with cortical microtubules are referred to as microtubule-associated cellulose synthase compartments (MASCs) [35]. CSI1 is apparently the molecular linker between CSCs and cortical microtubules, since loss of CSI1 induces disassociation of CSCs from microtubules and results in cellulose deficiency [39]. CSI1 apparently connects CSCs to microtubules through the CC1/2 N-terminal domain, which also influences microtubule stability under salt stress [36, 40]. Tagged CSI1 protein labels both SmaCCs and MASCs, suggesting that it might be required for both CSC delivery and recycling [33]. While CBIs like ISX or thaxtomin A induce CSC internalization, other CBIs like dichlobenil inhibit CESA motility and induce localized accumulation at the plasma membrane [41]. The differential effects of CBIs on CESA dynamics are useful tools to dissect cellular processes affecting CSC activity. Even though certain ISX-induced responses (gene expression changes, JA/SA and lignin accumulation) can be prevented by osmoticum co-treatments (sorbitol or mannitol application), CESA internalization cannot be prevented by these treatments [42]. This suggests that CSC displacement itself is not the stimulus responsible for osmo-sensitive responses activated by the CWI maintenance mechanism.

The actin cytoskeleton has also been implicated in cellulose biosynthesis, since actin manipulation leads to cellulose deficiency and defective CESA trafficking [43]. This led to the hypothesis that the actin cytoskeleton mediates delivery of CESAs to the PM, where CSC adaptors interact with the CSCs and cortical microtubules to ensure correct CSC positioning in the PM. Actin cytoskeleton dynamics seem to respond differentially to different types of stress, since plasmolysis induces thicker actin bundles while CBI application leads to actin filament disruption [44]. In addition to actin and microtubules both motor proteins and cytoskeleton-associated proteins have been implicated in cellulose biosynthesis and response to stress. Loss of the Arabidopsis kinesin FRAGILE FIBER1 (FRA1) alters cellulose microfibril organization, implicating kinesins in CESA trafficking and/or positioning [45, 46]. Similarly, other CSC/cytoskeleton interacting proteins like the endo (1 → 4) β-d-glucanase KORRIGAN1 (KOR1), CHITINASE-LIKE 1 (CTL1/POM1) and CTL2 have been implicated in cellulose synthesis [47–49] (Fig. 1a). KOR1 was found to be associated with primary CESAs and seems required for both glucan chain synthesis and regulation of intracellular CSC dynamics [50, 51], while absence of CTL1/POM1 and CTL2 impairs motility of CSCs. Similar to cellulose mutants *ctl1* seedlings exhibit both root cell swelling and ectopic lignin deposition [49, 52, 53]. It has been reported that the expression of *CTL1* is controlled by GENERAL CONTROL NON‐REPRESSED PROTEIN *5* (*GCN5*) which encodes a histone acetyltransferase [54]. Plants lacking GCN5 exhibit reduced cellulose levels, salt and ISX-hypersensitivity, providing further evidence that GCN5 is involved in regulating cell wall metabolism. These recent observations are exciting since epigenetic control has not been associated before with the regulation of CWI and salt stress responses.

CSCs are also required for cellulose biosynthesis during secondary cell wall formation, where they consist of AtCESA 4, 7 and 8 [55] (Fig. 1a). As in the case of primary cell wall CESAs, secondary cell wall CESAs were identified through genetic screens that identified irregular xylem (*irx*) mutants (AtCESA8^*irx1*^, AtCESA7^*irx3*^ and AtCESA4^*irx5*^), illustrating the importance of cellulose in stem development and stability [13, 15]. By creating catalytically inactive isoforms of the three CESAs, their individual activities during cellulose biosynthesis in developing secondary cell walls were determined [56]. Intriguingly, KO alleles for At*CESA4*, *7* and *8* do not cause lethality, thus allowing to study the effects of reduced cellulose production on pathogen responses. Interestingly, At*CESA*8^*irx1*^, At*CESA7*^*irx3*^, and At*CESA4*^*irx5*^ confer resistance to *Ralstonia solanacearum* and *Plectosphaerella cucumerina* [57]. The resistance seems to be mediated by abscisic acid (ABA) signaling being constitutively activated by the defects in secondary cell wall formation [57]. This is in contrast to the production of SA and JA in response to primary cellulose inhibition, suggesting that differences in the responses to cellulose impairment exist between primary and secondary cell walls [24, 29].

### Cellulose biosynthesis in monocots

While similarities exist regarding the molecular components involved in cellulose biosynthesis in mono and dicots, there are also differences. All dicots and most monocots have type I cell walls while species belonging to the *Poaceae* family have type II cell walls [58]. Many cultivated crops such as maize, rice (*Oryza sativa L*.) and wheat belong to the *Poaceae* family. Both type I and type II cell walls contain cellulose produced by cellulose synthases of the CESA gene family [59]. However, in contrast to type I walls, pectins contribute only to a small extent to the matrix polymers in type II walls. Moreover, in type II cell walls, cellulose microfibrils are crosslinked by glucuronoarabinoxylans instead of hemicellulose-pectin as in type I cell walls [58].

In monocot species, like barley (*Hordeum vulgare,* HvCESA), rice (*Oryza sativa,* OsCESA) and mais (*Zea mays,* ZmCESA), CESAs have been identified primarily based on homologies to Arabidopsis CESA genes [60]. In parallel brittle culm (*bc*) mutants were isolated, which exhibit reduced amounts of cellulose and decreased breaking strength [61, 62]. The rice brittle culm mutant *S1*-*24* consists of a point mutation in Os*CESA7*, which causes reduced cellulose production in sclerenchyma cells thus leading to lower mechanical strength in the sclerenchyma cell walls [63]. Another *bc* mutant, *bc5*, leads to cell wall formation defects in sclerenchyma nodes, likely due to altered expression of Os*CESA4, 7* and *9* as well as reductions in cellulose and hemicellulose production [64]. In rice and *Sorghum bicolor*, *bc1* mutations have been recently shown to reside in genes encoding homologues of Arabidopsis COBRA-like proteins [65, 66] (Fig. 1a, b). COBRA-like proteins are GPI-anchored proteins, which are required for cellulose biosynthesis both during primary and secondary cell wall formation [66, 67]. Just like in At*cobl4* secondary cell walls, cellulose content is altered in both Sb*bc1* and Os*bc1*, causing increased stem brittleness [65, 66]. Similar to Arabidopsis *FRA1*, rice FRA1 homologue (Os*BC12*) has been associated with orientation of cellulose microfibrils [68]. The existing evidence indicates pronounced similarities regarding the mode of action of cellulose biosynthesis in monocots and dicots, but our knowledge regarding the presence of a CWI maintenance mechanism in monocots is very limited. For example, treatment of *Poa annua* with the CBI Indaziflam inhibits growth, triggers root cell wall swelling and ectopic lignification, responses previously observed upon ISX treatment in dicots [69]. More targeted research activities will be required to assess similarities and differences of CBI responses in dicots and monocots.

### Cellulose synthase-like enzymes in monocots and dicots

Cellulose synthase-like enzymes (CSLs) are divided into 9 subfamilies (CSLA–H and CSLJ), all belonging to the large glycosyltransferase 2 (GT2) superfamily [70, 71]. Comparative genome studies suggest that several of these families are restricted to dicots, like *CSLG*, while others, like *CSLF* and *CSLH* seem restricted to grass species [71]. Members of the *CSLA*, *CSLC*, and *CSLD* families have been identified in all land plants where genome sequence information is available, including the moss *Physcomitrella patens* and the lycophyte *Selaginella moellendorffii* [72, 73]. Based on genome sequence analysis, a super family of CSL proteins, grouped in 6 families named from CSLA–G (no F), has been identified in Arabidopsis [71]. Several mutants have been isolated in Arabidopsis, but only a small number of them exhibit pronounced phenotypes, which are not associated with cellulose biosynthesis. CSLD mutants such as *csld2* and *csld3* (the latter known as *kojak*) display defective root hair development in primary roots and *csld2/csld3/csld5* plants exhibit reduced growth, suggesting the three proteins might be part of a multimeric complex [74–76]. Members of the CSLA and CSLD families have been implicated in the synthesis of mannans and glucomannans, while members of the CSLC family are required for synthesis of xyloglucans and other polysaccharides related to hemicellulose synthesis [75–77]. Intriguingly, the *csld3* phenotype is rescued by expressing a modified version of CSLD3 protein containing the CESA6 catalytic domain, which suggests functional redundancy between CSL proteins and primary CESAs [78]. Mutants have been isolated for several members of the CSLA family, providing insights into their respective biological and biochemical functions. In Arabidopsis, mutations in *CSLA9* (encoding a glucomannan synthase) cause reduced lateral root formation while mutations in *CSLA7* cause embryo lethality [79, 80]. Notably, the *csla*7 phenotype can be rescued by overexpressing *CSLA9*, suggesting that *CSLA*7 and *CSLA9* have similar biochemical functions and differences in activity might be caused by differential gene expression [80]. In Arabidopsis, mannans are present at low levels in primary cell walls and in greater abundance in secondary cell walls [81–83]. Arabidopsis *csla2/csla3/csla9* triple mutants, lacking detectable glucomannans in stems, do not show alterations in stem development and structure [80]. However, if glucomannan content is enhanced (by over expressing CSLAs) plant development is affected [80]. This suggests that glucomannans are not growth limiting cell wall components and their absence can be compensated. However, increasing their levels has significant consequences, with the reasons to be determined.

A major difference between Arabidopsis and grass cell wall metabolism can be found in the functions of the CSLs, likely reflecting their role in the synthesis of monocot specific cell wall saccharides [84]. In fact, several CSLs are involved in the synthesis of mixed-linkage glucans (MLGs) and act as (1 → 3)/(1 → 4)-β-glucan synthases. The difference is further illustrated by the fact that in rice the CESA/CSLs superfamily has 45 members [85]. MLGs have been detected in large amounts in endosperms and therefore a storage function was proposed for them [86]. In maize and barley coleoptiles they are present in elongating tissues, while in several other grass species (including rice, *Miscanthus* and *B. distachyon*) they have been also observed in dry stems [87]. These observations suggest that MLGs are incorporated both into primary and secondary cell walls. Members of the CSLF family in rice as well as of the CSLH and J families in barley are involved in the biosynthesis of MLGs [71, 88]. Loss of function alleles of rice *CSLF6* (*cslf6*-*1* and *cslf6*-*2)* exhibit a 99% reduction of MLGs in developing leaves, while mature stems have weaker and more brittle cell walls, but mutant plants were able to grow [89] (Fig. 1b). This is surprising since these MLGs were supposed to have a function in type II cell walls similar to pectins in type I cell walls, i.e., forming a gel-like matrix in which cellulose microfibrils are embedded [90–92]. However, detailed analysis of cell wall composition found that *cslf6*-*1* and *cslf6*-*2* mutants exhibit moderate reduction in cellulose content compared to wildtype controls and suggested that MLGs may influence the spatial distribution of cellulose microfibrils [89, 93]. These observations also imply that manipulation of MLG production affect cell wall organisation, with the resulting phenotypes not easily explained by functional redundancies, supporting the existence of a CWI maintenance mechanism in these plant species.

## Hemicellulose metabolism and effects of its impairment

Historically, non-cellulosic and non-pectic polysaccharides have been collectively named hemicelluloses. The classic features of hemicelluloses are β (1 → 4) glycosidic bonds, which form the dominant backbone linkage in an equatorial configuration [5]. Following this classification only a limited number of polymers (xyloglucan, xylans, mannans, glucomannans, and MLGs that are restricted to different grass families) will be discussed here with mannan and MLG biosynthesis being excluded since these topics have been covered in the previous section.

### Xyloglucan in primary cell walls of dicots

Xyloglucan (XyG) consists of β (1 → 4)-linked glucans sometimes partially substituted with xylosyl substituents at the O-6 position or with species-specific side chain decorations. In graminaceous monocots, xylans and MLGs form major components of both primary and secondary cell walls [94]. While xyloglucan (XyG) comprises only 2–5% of the primary cell wall in monocots, it is the most abundant hemicellulose of primary cell walls in dicots (10–20%) and a minor component of secondary cell walls [90, 94–96]. Interestingly, in dicots XyG establishes interactions between cellulose microfibrils and pectins [97]. XyG turnover seems to influence cell elongation and cell wall remodeling [98]. In Arabidopsis, these events are mediated by XyG endotransglucosylases/hydrolases, (belonging to CAZy family GH16) [99, 100]. Similar to pectin and cellulose fragments, XyG breakdown products play a role in signaling in different plant species [101–103]. The complexity of XyG biosynthesis and sidechains means that many different enzymes are required for the synthesis of glucan chains and xylosyl, galactosyl or fucosyl substitutions. Members of the CSLC family (like CSLC4, CSLC5, and CSLC6) act as β (1 → 4)glucan synthases (using UDP-glucose as substrate) contributing to the production of the XyG backbone [104] (Fig. 1a). Xylosyl substitution is carried out by XyG xylosyltransferases (XXTs), which in Arabidopsis belong to glycosyltransferase (GT) family 34 and are exemplified by XXT1 and XXT2 [96]. *xxt1xxt2* seedlings display aberrant and swollen root hairs and shorter hypocotyls. Adult *xxt1xxt2* plants lack detectable XyG and display mild growth phenotypes characterized by reduced rosette size and shorter petioles with enhanced mechanical extensibility compared to wildtype controls, indicating a role for XyG in modulating cell wall strength [105]. These rather mild mutant phenotypes raise questions regarding the importance of XyG for plant growth under standard growth conditions [96]. XXT activity seems highly conserved, since expression of Os*XXT1* in Arabidopsis rescues the root hair defects of Arabidopsis *xxt1xxt2* plants [106]. In Arabidopsis, gene expression analysis showed that *XXT1* and *CSLC4* are expressed in the same tissues and developmental stages, suggesting that XXT1 and CSLC4 might be both active in glucan synthesis. Moreover, XXT1, XXT2, XXT5 and CSLC4 seem to be localized in the Golgi, where they add xylosyl groups to the glucan backbone [104] (Fig. 1a). XYLOGLUCAN L-SIDECHAIN GALACTOSYLTRANSFERASE 2 (XLT2) and MURUS3 (MUR3) were both shown to act as galactosyltransferases in Arabidopsis [107, 108]. They are required for adding d‐galactopyranosyl (β-d-Galp) to an internal d‐xylopyranosyl (α-d-Xylp) or an α-d-Xylp adjacent to an unbranched β-d-Glc. Interestingly, *MUR3* KO plants (*mur3*-*3*) exhibit dramatically reduced growth, display cabbage-like leaves and are constitutively resistant to *Hyaloperonospora parasitica* infections [107, 109]. XyG in *mur3* is characterized by reduced levels of galactosyl substitutions and almost no fucosyl residues. XXTs and MUR3 might have tissue-specific functions, because *mur3* does not exhibit root hair phenotypes as observed in *xxt1 xxt2* [108, 110]. Interestingly, *MUR3* mutant phenotypes are rescued by knocking out xylosyltransferases (such as *XXT1*, *2* and *5*) [107]. Double and triple mutant plants (*xxt1/xxt2/mur3*-*3, xxt2/mur3*-*3* and *xxt5/mur3*-*3)* resemble wildtype plants both with respect to growth phenotypes and on the transcriptomic level, even though XyG deficiency is not reversed in the double or triple mutant backgrounds [111]. As in the case of glucomannans it seems that plants can adapt to the absence of XyG, while dysfunctional XyG structures have deleterious effects [107]. Intriguingly, plants lacking *XLT2* are phenotypically normal, lacking the cabbage-like phenotype of *mur3* plants [107]. This implies that the severe phenotypes are caused by specific changes in galactosylation of XyG mediated by MUR3 and that these have a biological function and/or their presence is detected by the plant causing responses [107]. Since MUR3, like XXT1, localizes to the Golgi cis and medial *cisternae* (Fig. 1a), the existence of a dedicated XyG synthesis complex has been proposed [112]. The biological role of XyG fucosylation remains to be fully determined since l-fucosylated oligosaccharins inhibit pea epicotyls elongation and fucosylated XyG bind cellulose in vitro even though cellulose–xyloglucan binding does not require XyG fucosylation [113, 114]. These observations indicate that plants can recognize specific changes in XyG structures, compensate for deviations from the normal state and that the effects are specific as illustrated by differences between XLT2 and MUR3. However, we do not know the stimuli indicating deviations, the detection mechanism in place or the mode of action of the downstream processes bringing about changes in other cell wall polysaccharides.

### Xylan formation and modification in monocots and dicots

Xylans include all the hemicellulose polymers containing (l → 4)-linked β-d-xylopyranosyl units as backbone, like glucuronoxylan, arabinoxylan, glucuronoarabinoxylan, and l-arabino-(4-*O*-methyl-d-glucurono)-xylan. Xylans are important components of cell walls and produced by a group of enzymes [115]. Roughly 10–20% of the xylose sugars in the xylan backbone are substituted. Xylan side chains frequently contain arabinose, which either forms a single substituent or connects other sugars like xylose, galactose and 4-*O*-methyl-d-glucuronic acid to the xylan backbone [116]. In contrast, methylated glucuronic acid (GlcA) can directly bind xylose, without the need for an arabinose linker [5]. In Arabidopsis the degree of polymerization (DP) of xylan in secondary cell walls can reach up to 150 xylose units while in grasses (like wheat) the DP can reach up to 4000 xylose repetitions [117]. In grasses, xylans frequently exhibit arabinose side chains and are extensively decorated with α-(1 → 2)- and α-(1 → 3)-linked arabinofuranose molecules. Moreover, in these plants xylans are crosslinked via ferulic acid residues to lignin [5]. Even though xylans are the second most abundant biopolymers after cellulose, xylan biosynthesis is still poorly understood at the biochemical level. In Arabidopsis, a family of GT43 glycosyltransferases (IRX9/IRX9-L, IRX14/IRX14-L) has been implicated in the synthesis of xylan backbones during secondary cell wall formation [118, 119] (Fig. 1a). In monocots (specifically *Poales*), glucuronoarabinoxylan is the main hemicellulose in primary cell walls while xylan side chains interact with lignin in secondary cell walls to enhance cell wall strength [120, 121]. In the rice genome, ten *GT43* encoding genes were identified but the involvement in xylan biosynthesis has not been confirmed for all of them yet [116]. Similarly to XyG, xylan biosynthesis seems to also occur in the Golgi based on localization data for OsGT43 proteins [116]. In Arabidopsis, both IRREGULAR XYLEM9 and 14 (IRX9, IRX14) have xylosyltransferase activity and are required to extend xylan backbones [122]. *IRX9* and *IRX14* mutant plants are slightly smaller than wildtype controls, exhibit thinner xylem vessel cell walls and have reduced levels of xylose [123]. IRX9-LIKE (IRX9-L) and IRX14-LIKE (IRX14-L), respective homologues of IRX9 and IRX14, are also members of the GT43 family and loss of function alleles result in no obvious mutant phenotypes [124]. However, *irx9*-*2/irx9*-*L* and *irx14/irx14*-*L* plants exhibit stunted growth with small leaves and reduced xylan levels [124]. Heterologous expression of the rice Os*GT43A* and Os*GT43E* in At*irx9* or Os*GT43J* in At*irx14* rescues the mutant phenotypes, indicating that OsGT43A and OsGT43E represent rice homologs of the Arabidopsis genes and that their enzymatic activities are conserved [116]. Glucuronoxylan is made of a backbone of β (l → 4)-linked xylose residues decorated with α (1 → 2)-linked GlcA or 4-*O*-methyl-GlcA (MeGlcA) [125]. IRX10 and IRX10-L, belonging to the Arabidopsis GT47 family, are required for its synthesis [126, 127] (Fig. 1a). However, while *irx10* and *irx10*-*l* single mutants are phenotypically normal, suggesting functional redundancy between the GT47 family members, *irx10/irx10*-*l* double mutants are severely dwarfed, exhibit thinner cell walls in xylem vessels and produce infertile flowers [126]. The results suggest that for certain types of cell wall modifications either no functional redundancy exists or no CWI monitoring mechanism is in place to initiate compensatory responses.

Bioinformatics-based analyses of grass genomes suggest that GT47 and GT61 glycosyltransferase families may encode xylan arabinosyltransferases (XATs) [128]. Heterologous expression of either wheat or rice GT61s in Arabidopsis leads to mono-arabinosylation of xylan chains, supporting a role for GT61s as α-(1 → 3)-arabinosyltransferases [128]. Phenotypes observed in wheat (*Triticum aestivum*) where Ta*XAT1* expression was suppressed by RNAi suggest that TaXAT1 mediates α-(1 → 3)-arabinose mono substitution of xylose backbones [129]. Other members of the GT61 family (like rice OsXAT2, OsXAT3 and wheat TaXAT2) function also as arabinosyltransferases, since they complement mutant phenotypes of homologous Arabidopsis genes [128]. In addition, it was shown that the OsXYXT1 function as xylosyltransferase [130]. Intriguingly, a forward genetic screen for mutations affecting saccharification performance in *Brachypodium distachyon* identified a mutation, which seems to reside in a GT61 family member and improves saccharification performance of the plant material while not having obvious impacts on plant growth and interaction with the environment [131]. Members of the GT8 family function as glucuronyltransferases. For α (1 → 2)-linked GlcA SUBSTITUTION OF XYLAN 1, 2 and 4 (GUX1, 2, 4), xylan glucuronosyltransferase activities have been demonstrated [132]. However, even if *gux1 gux2* plants show reduced glucuronoxylan levels, they do not exhibit growth defects. At the same time, *gux1/gux2/gux3* triple mutant plants are smaller than controls and exhibit reduced resistance to breaking force in stems [133]. In the triple mutant plants, interaction between xylan and cellulose seems impaired, leading to reduction in secondary cell wall thickness and mechanical strength [125].

Glucuronoarabinoxylans have been detected in rice and other monocots in large amounts in both primary and secondary cell walls and are modified in different ways [134]. Examples for such modifications include acetylation and methylation. In non-woody plants 30% of GlcA is not methylated while in woody plants nearly all of the GlcA is [135]. The degree of glucuronoarabinoxylan methylation together with the amount of arabinose substitutions differs between monocot species and can also vary between tissues from the same species [136]. An important structural component of glucuronoarabinoxylans in grasses is the presence of ferulic and coumaric acid, which is thought to provide additional cell wall strength [137]. In dicots, glucuronoxylan methylation is carried out by DUF579 domain-containing proteins, like the GLUCURONOXYLAN METHYLTRANSFERASE1 (GXMT1) [138]. *gxmt1*-*1* mutants show reduced glucuronoxylan methylation coupled with enhanced lignin methylation [138]. Interestingly, no mechanical defects are observed in their stems suggesting that either changes in methylation do not affect the mechanical cell wall properties or the effects of their modification are neutralized. Acetylation is performed by AXY9 (ALTERED XYLOGLUCAN9), *REDUCED WALL ACETYLATION* (RWA) and DUF231 domain-containing proteins [139–142]. Mutations in the four RWAs reduce stem resistance to mechanical force, suggesting that xylan acetylation modulates mechanical stability. Our current knowledge regarding glucuronoxylan formation and modification is still rather limited as evidenced by the overview provided here. However, combining bioinformatics studies with heterologous expressions in Arabidopsis will help to fill in the knowledge gaps. In several cases, exemplified by IRX9/IRX9-L, IRX10/IRX10-L IRX14/IRX14-L and GUX1, 2, 3, the results obtained suggest that functional redundancy between enzymes with similar activities is responsible for the mutant phenotypes observed. In other cases like the methylation of glucuronoxylan, the corresponding changes observed in lignin methylation suggest adaptations in cell wall metabolism take place, but the mode of action of the mechanism responsible remains to be determined. To summarize, with respect to CWI maintenance and hemicelluloses, there is currently no clear evidence that hemicellulose integrity is monitored in a similar manner as described for cellulose. This might indicate either the absence of dedicated monitoring mechanisms or simply a lack of knowledge due to limited research activity in the area.

## Pectin metabolism and effects of its impairment

While cellulose and hemicellulose are major components of load-bearing polysaccharide networks in both primary and secondary cell walls, pectins are mostly found in primary walls and play a role in the context of development, growth and adaptation to changing environments. Biochemically, pectins are usually defined as galacturonic acid (GalA)-containing polysaccharides [143]. Homogalacturonan (HG) is a polysaccharide consisting of α-(l → 4)-linked GalA residues and is covalently bound to RG-I and RG-II [143]. RG-II consists of a GalA backbone decorated with complex, yet conserved, side chains that contribute to cell adhesion and rigidity by complexing borate as dimer [143]. The RG-I backbone consists of alternating GalA and rhamnose residues, decorated with arabinan, galactan and arabinogalactan side chains that differ depending on species and developmental stage [143].

Pectins are major components of the early cell plate during cytokinesis and primary cell walls, where they contribute to the regulation of cell-to-cell adhesion and cell elongation [144, 145]. Seeds of flowering plants produce pectin-rich mucilage, which is important for seed hydration, germination and adherence [146]. Regulation of these different processes is attributed to pectin methylesterases (PMEs), which control the degree of methylesterification (DM) in HG and influence HG cross-linking through Ca^2+^-bridges [147]. The controlled modification and degradation of pectin is crucial for cell separation processes, such as leaf abscission and fruit ripening [144, 148]. While pectins have previously been regarded as a gel, in which a hemicellulose-tethered cellulose scaffold is embedded, more recent research indicated that direct pectin-cellulose interactions exist [149]. It is currently not clear how this interaction works and which pectin polymers are involved, questions that have important implications for potential load-bearing functions of pectin. Compared to other cell wall components, pectin seems to be more readily degraded upon environmental stress. Examples are pathogen-induced breakdown of pectin enhancing infection success [150] or mobilization of carbohydrates from pectin during starvation periods [151]. Interestingly both intact pectin and pectin fragments have been shown to bind to plasma membrane-localized receptor kinases, suggesting that pectin integrity is closely monitored by plant cells [152, 153].

### Pectin in primary cell walls of dicots

Pectin polymers are formed in the Golgi from nucleotide sugars. While the chemical complexity of the different pectin polymers implicates a large number of transferases in pectin formation, only few transferase proteins have been characterized in detail. HG is synthesized by GALACTURONOSYLTRANSFERASE1 (GAUT1), which is anchored to the Golgi by GAUT7 [154, 155] (Fig. 1a). The GAUT1:GAUT7 complex seems to include, or associate with, several additional cell wall biosynthesis proteins, such as the putative HG- methyltransferase QUASIMODO3 (QUA3) and KOR1 [155, 156]. GAUT8/QUA1 is required for HG biosynthesis and cell adhesion [157, 158] but also for normal xylan biosynthesis [159]. Interestingly, loss of putative HG-methyltransferases like QUA2 and GAUT8/QUA1 causes similar mutant phenotypes including reduced HG content, impaired cell adhesion and stunted growth [160]. Similarly, the QUA2-unrelated putative methyltransferases COTTON GOLGI-RELATED2 (CGR2) and CGR3 are required for normal pectin composition and growth, further highlighting the importance of pectin modification for plant development [161]. A recent proteomic analysis, characterizing auxin-dependent alterations in root tissue, identified GAUT10 as positive regulator of the root apical meristem length and cell number in a process that seems to be sucrose-sensitive [162]. CWD on the other hand negatively affects root apical meristem length and cell number, emphasizing the importance of CWI maintenance for root development [42]. Xylosylation of HG is mediated by XYLOGALACTURONAN DEFICIENT1 (XGD1), but the biological function of xylogalacturonan remains to be determined [163] (Fig. 1a). While the transferases involved in RG-I backbone syntheses are unknown, the synthesis of RG-I side chains is understood much better. β-(l → 4)-galactan is synthesized by GALACTAN SYNTHASE1 (GALS1) and putatively its two homologs, GALS2 and GALS3, which exhibit weaker overall expression and distinctly specific expression patterns [164]. ARABINAN DEFICIENT1 (ARAD1) and ARAD2 have been identified as non-redundant putative α-(l → 5)-arabinosyltransferases, which seem to form a complex at least during transient overexpression [165, 166]. Surprisingly, despite their impact on RG-I side chain abundance, no growth phenotypes have been reported for *gals1* and *arad1/arad2* mutant plants [164, 166]. Recently, GAUT11 and MUCILAGE-RELATED70 (MUCI70) were shown to be essential for RG-I production in seed mucilage [143, 167]. While the biochemical activity of MUCI70 is unknown, GAUT11 has been shown to have HG:GalA transferase activity, suggesting that HG and RG-I elongation in mucilage might require mutual enzyme activities [167]. The only transferase activity so far reported for RG-II synthesis is mediated by the RHAMNOGALACTURONAN XYLOSYLTRANSFERASEs (RGXTs), RGXT1, RGXT2, RGXT3 and RGXT4 [168–170]. While mutants for *RGXT1* and *RGXT2* show no obvious phenotypes and *rgxt3* mutants have not been studied yet, loss of *RGXT4* leads to defective pollen tube and root growth [168–170]. The decoration of RG-II with galactose can be reduced by RNA interference-induced suppression of *GDP*-*GALACTOSE TRANSPORTER1* (*GGLT1*) expression, leading to reduced RG-II dimerization and pronounced growth defects [171] (Fig. 1a). Impaired RG-II dimerization and growth have been previously described in *mur1*, where fucosylation of several polysaccharides is affected [172, 173]. Congruently, both *mur1* and *gglt1* mutants are impaired in growth recovery after salt stress [153, 171]. General growth defects in *mur1* and *gglt1* and root growth defects in *rgxt4* mutants can be rescued by borate supplementation, illustrating the importance of RG-II dimerization for cell wall function [171, 172]. Moreover, pectin-crosslinking by combined borate- and Ca^2+^-treatment can rescue impaired CWI during growth recovery after salt stress [153]. Similar to hemicellulose, HG and RG-I can be acetylated by members of the RWA and TRICHOME BIREFRINGENCE-LIKE (TBL) protein families [174]. While RWA2 is required for acetylation of both xyloglucan and pectin, TBL10 is specifically required for RG-I acetylation [142, 175]. Mutants for *TBL10* do not display a visible growth phenotype but exhibited increased drought stress tolerance [175]. Loss of TBL10 does not affect pathogen resistance, which is surprising, since overexpression of *Aspergillus nidulans* RG-I acetylesterase or loss of *RWA2* lead to increased resistance to the necrotrophic fungal pathogen *Botrytis cinerea* [142, 176]. TBL44/POWDERY MILDEW RESISTANT5 (PMR5) seems to acetylate HG and mutants are affected in cell wall acetylation, pectin and cellulose content [177, 178]. *pmr5* mutants exhibit increased resistance to biotrophic powdery mildew fungi and the hemibiotrophic fungus *Colletotrichum higginsianum* [151, 177]. Powdery mildew resistance of *pmr5* is partially suppressed by a mutation in *RWA2,* whereas *rwa2* plants are hypersusceptible to powdery mildew infection [178]. Hyphae from powdery mildew and *C. higginsianum* appeared deformed or shrunken on infected *pmr5* tissues, indicating that pectin modification is critical for host–pathogen compatibility and hyphal expansion. This defect in fungal growth is aggravated on *pmr5*/*pmr6* double mutant plants, which show increased pectin content (PMR6 encodes a putative pectate lyase) and penetration resistance to *C. higginsianum* [151, 177]. In contrast, *mur8* mutants, exhibiting reduced RG-I content in leaves, display strongly reduced penetration resistance to this fungus [151, 179].

The above-mentioned mutant phenotypes illustrate the impact of intact pectin biosynthesis and modification on CWI during growth, development and (a)biotic stress. Modification of the degree of acetylation and methylesterification is achieved by the activities of pectin acetyl esterases (PAEs) and PMEs, respectively. *PAE*s and *PME*s form large gene families and their functions have been reviewed in detail elsewhere [180]. Here, we will only present selected examples to highlight the importance of pectin acetylation and methylesterification for functional integrity of plant cell walls. Heterologous expression of a *Vigna radiata* PAE in potato tubers caused a strong decrease in pectin acetylation and increased stiffness of cell walls [181]. Overexpression of *Populus trichocarpa PECTIN ACETYLESTERASE1* (Pt*PAE1*) in tobacco caused defects in cell elongation of floral filaments and pollen tube growth, suggesting that regulation of pectin acetylation might be critical for plant reproduction [182]. PAE activity is dependent on the DM of HG and has direct implications for the formation of Ca^2+^ “egg box” structures, indicating synergism between PAEs and PMEs [180]. Similar to the pathogen response phenotypes reported for *rwa* and *tbl* mutants, several studies demonstrated pronounced effects of loss or overexpression of *PME* or *PME* inhibitor (*PMEI*) genes on resistance to bacterial, fungal and viral pathogens [183–188]. The DM is critical for regulation of cell wall elasticity and this is mechanistically achieved by the activities of PMEs and their endogenous inhibitors [189]. Research into the cell wall mechanics of Arabidopsis plants overexpressing PME5 or PMEI3 indicated that asymmetric de-methylesterification underlies growth symmetry breaking and anisotropic growth [145]. Apparently, loss or inhibition of PME activity can have a direct and severe impact on the mechanical strength of tissue, as reported for *pme35* mutant and *PMEI5* over-expressing plants [190, 191]. Growth defects are probably in large part caused by brassinosteroid (BR) signalling, which is activated by impaired pectin structure [191]. Activation of BR signalling is mediated by RECEPTOR-LIKE PROTEIN44 (RLP44), which directly interacts with the BR receptor BRASSINOSTEROID INSENSITIVE1 (BRI1) and its co-receptor BRI1-ASSOCIATED KINASE1 (BAK1) [192, 193]. RLP44 also promotes interaction of BAK1 with PHYTOSULFOKINE-RECEPTOR1 (PSKR1) to control xylem cell fate and switching between BR and PSK pathways seems to be regulated through RLP44 phosphorylation [193, 194]. It would be interesting to understand how biochemical (pectin methylesterification status) or functional (status of cell wall integrity) information is perceived by RLP44.

Sensing of alterations in pectin polymerisation and structure is better understood for a family of RECEPTOR-LIKE KINASEs (RLKs), the WALL-ASSOCIATED KINASEs (WAKs). WAK1 and WAK2 bind to pectin oligomers in vitro, with a preference for de-esterified and short oligogalacturonides (OGs) [152]. The available literature on WAKs was reviewed recently and a model suggested, in which WAK-mediated signalling is modulated by the degree of HG polymerisation (intact pectin vs. OGs), thus representing a monitoring mechanism capable of detecting the extent of pectin degradation [152].

Using an elegant bioengineering approach, Benedetti et al. demonstrated that polygalacturonase-dependent in planta production of OGs induces defence responses and provides increased resistance to bacterial and fungal pathogens [195]. Since growth and development are negatively regulated by OG production, authors from the same lab investigated a regulatory mechanism inactivating OG-dependent signalling. The discovery of oxidized OGs by mass spectrometry lead them to the identification of OG OXIDASE1 (OGOX1), a berberine bridge enzyme-like protein, possibly required for regulation of growth-defence trade-off [196]. Recently, the characterization of in vivo produced OGs during *Botrytis cinerea* infection was accomplished [197]. Unexpectedly, OGs were mostly generated by the activity of fungal pectin lyases, yielding acetyl- and methylesterified OGs, instead of un-methylesterified OGs derived from polygalacturonase activity [197]. Polygalacturonase-dependent OGs did not occur in a degree of polymerization previously found to be elicitor-active, but only in shorter fragments that were oxidized late in the infection process [197]. Responses induced by elicitor-active un-methylesterified OGs seem to be distinct from responses induced through the osmo-sensitive CWI maintenance mechanism [198]. The latter causes JA accumulation in response to enzymatic degradation of Arabidopsis cell walls by polygalacturonase, a response absent in OG-treated Arabidopsis seedlings. Intriguingly, CWI impairment in Arabidopsis seedlings caused by CBI is correlated with increased cell wall pectin content, suggesting that pectin might accumulate to compensate for loss of cellulose in primary cell walls [199, 200].

**Table 2:** Proteins involved in CWI maintenance in dicotyledonous plants

Name	Mutant	Functions and mutant phenotypes	Reference
RECEPTOR-LIKE PROTEIN KINASE THESEUS 1	*the1*	Member of the CrRLK1L family. Required for ISX-induced CWD JA/SA/lignin. KO alleles partially suppress *prc1* phenotypes. THE1 binds RALF34	[198, 233, 291]
RECEPTOR-LIKE PROTEIN KINASE FERONIA	*fer*-*4*	Member of the CrRLK1L family. May act as pectin integrity sensor. KO plants have dramatic growth defects and pollinations is reduced. Required for response to RALF22 and 23. KO mutants show enhanced responses to ISX-induced CWD	[153, 233]
RECEPTOR-LIKE PROTEIN KINASE HERCULES1	*herk1*	Member of the CrRLK1L family. Single mutants are phenotypically normal. *herk1the1*-*4* plants show stunted growth, cell elongation defects *and* enhanced responses to ISX-induced CWD (*the1*-*4* is a gain of function allele)	[198, 292]
RECEPTOR-LIKE PROTEIN KINASE HERCULES2	*herk2*	Member of the CrRLK1L family. Single mutants are phenotypically normal. Enhanced responses to ISX-induced CWD	[198, 292]
RECEPTOR-LIKE PROTEIN KINASE ERULUS	*eru*	Member of the CrRLK1L family. Involved in root hair growth. KO mutants show reduced root hair length. Plants are characterized by reduced XyG content	[198, 293]
LRR RECEPTOR-LIKE KINASE	*fei2*	Single KO mutants show no obvious growth phenotype and reduced responses to ISX-induced CWD. Inhibition of FEI1 and 2 affects root length and cell expansion in response to high concentration of sucrose. Double mutants show cellulose deficiency and anisotropic cell expansion via ACC-mediated pathway	[198, 294]
MID1-COMPLEMENTING ACTIVITY 1	*mca1*	Encodes a stretch-activated Ca^++^channel. Complements mid1 yeast mutant and is required for activating Calcium-dependent CWD-induced signalling pathway. Adult plants are slightly smaller. Required to generate Calcium spikes in response to cold shock, *mca1* roots fail to penetrate hard agar medium and *mca1 mca2* plants exhibit enhanced sensitivity to cold stress	[198, 222, 295]
MID1-COMPLEMENTING ACTIVITY 2	*mca2*	*mca2* KO plants have no growth phenotypes. *mca1 mca2* plants are significatively smaller (rosette size and stem length)	[222]
MECHANOSENSITIVE ION CHANNEL PROTEIN 2	*msl2*	Chloroplast-localized mechanosensitive ion channel and homologue of MSL3. Mutant plants exhibit reduced rosette size	[296]
MECHANOSENSITIVE ION CHANNEL PROTEIN 3	*msl3*	Homologue of MSL2. *msl2 msl3* seedlings exhibit reduced responses to ISX-induced CWD, reduced plant size, chloroplasts with multiple Z-rings and variegated leaves due to chloroplast defects, chloroplasts in *msl2 msl3* seedlings exhibit problems with adaptation to osmotic stress	[198, 223, 224, 297]
NITRATE REDUCTASE 1	*nia1*	KO plants show normal development	
NITRATE REDUCTASE 2	*nia2*	KO plants show normal development *nia1 nia2* plants show smaller rosettes and exhibit significantly reduced responses to ISX-induced CWD	[42, 198, 298]
WALL STRESS RESPONSE 1/DIRIGENT-LIKE PROTEIN 7	*wsr1/dir7*	KO plants show normal development and reduced responses to CBI-induced CWD (reduced SA levels) and enhanced *Plectosphaerella cucumerina* susceptibility	[238]
WALL STRESS RESPONSE 4/PXY/TDR-CORRELATED 3	*wsr4/pxc3*	KO plants show normal development. Reduced cellulose content in stem and leaves. Reduced responses to CBI-induced CWD (JA and lignification)	[238]

### Pectin in monocots

Pectins are generally less abundant in monocot species compared to dicots and particularly lowly abundant in grasses [120]. Nonetheless, interesting phenotypes have been reported for mutants affected in pectin biosynthesis or modification. Downregulation of the HG:GalA transferase GAUT4 in switchgrass leads to reduced HG and RG-II content, increased growth and cell wall extractability, thus improving properties for biofuel production from lignocellulosic biomass [201]. Further research indicated that reduced recalcitrance to cell wall extraction also stems from alterations in hemicellulose and lignin structure. In particular, reduced lignin-carbohydrate complex cross-linkages seem to facilitate lignin degradation during pre-treatment of lignocellulosic biomass [202].

PME activity has been demonstrated to be of crucial importance in grasses during development and for pathogen resistance. For example, recent research showed that pollen- and pistil-expressed PMEs control cross-incompatibility in maize [203, 204]. Similar to findings in dicots, modulation of PME activity by host plants or fungal pathogens contributes to the outcome of plant-pathogen interaction in wheat, demonstrating the importance of HG DM despite the low overall abundance of pectin in cell walls of grasses [205, 206]. Pectin degradation induced by overexpression of the polygalacturonase subunit *OsBURP16* in rice-impaired cell adhesion and increased sensitivity to cold, salinity and drought stress, indicating a critical role of intact pectin structure for abiotic stress tolerance [207]. Aluminum toxicity represents another economically important cause of abiotic stress that is strongly influenced by pectin abundance and modification. The DM of pectin determines the binding capacity of root cell walls for aluminum in maize, rice and wheat [208–210]. In wheat, exposure to aluminum induces nitric oxide accumulation, which in turn leads to increased PME activity and a reduced DM of pectin [210]. While no direct role for nitric oxide in CWI signaling has been shown to date, recent evidence in Arabidopsis supported the idea that nitrate reductase-dependent nitric oxide formation might be required for the initiation of cell wall damage responses [42]. These representative examples illustrate that even if a cell wall compound like pectin in monocot cell walls is only lowly abundant it still can have profound impact on cell wall composition, structure and performance during growth, development, interaction with the environment and economically relevant feedstock traits. It also reinforces the question how changes in cell wall structure and composition are detected and the resulting changes brought about.

## The plant cell wall integrity maintenance mechanism in dicots

The overview provided above illustrates comprehensively the plasticity of plant cell walls and how their composition and structure change in highly dynamic ways in different plant species. The changes, induced by developmental cues, interactions with the environment or genetic manipulation, ensure that cell walls can perform their biological functions in different situations. In addition to these functions, CWI has to be maintained constantly as well since otherwise the high turgor pressure levels, prevalent in plant cells, will lead to cell bursting and death. The available evidence, deriving often from research on primary cell walls in dicots, supports the existence of a dedicated plant CWI maintenance mechanism [191, 211, 212]. This mechanism is monitoring the functional integrity of cell walls and initiating adaptive changes in cell wall and cellular metabolism to maintain integrity. Impairment of CWI can occur during growth and development, interaction between plants and their environment (including exposure to biotic and abiotic stress) and also through artificial measures like inhibitor treatments (CBIs) or genetic manipulation of cell wall metabolism. The extent of impairment probably varies, ranging from increased strain during etiolation in elongating hypocotyls to physical CWD and cell wall rupture during exposure to abiotic or biotic stress like drought, high salinity or pathogen infection [150, 213, 214]. Here, we will focus on the knowledge available regarding CWI maintenance in primary cell walls, discuss how CWI impairment may be perceived in plant cells and review how CWI signalling is integrated with other processes to regulate downstream responses exemplified by integration of CWI signalling with signals deriving from pattern triggered immunity (PTI). We will not incorporate the available knowledge regarding CWI maintenance in pollen tube growth since this has been recently reviewed comprehensively [215].

The existence of a CWI maintenance mechanism in plants was postulated after observing that reduction in cellulose production in the root elongation zone of Arabidopsis seedlings either by genetic means or CBI treatment leads to ectopic deposition of lignin (as a putatively compensatory measure) and activation of defence responses (including production of JA and induction of defence genes like *VSP1*, *PDF1.2*) [24, 26–28]. Follow-up studies showed that CBI-induced changes in cell wall composition also include enhanced callose production and modifications of neutral cell wall sugars [199, 200]. When cell wall-degrading enzymes (like pectinase and cellulase) are used to induce CWD, lignin and callose deposition are also detectable in cotyledons, suggesting that the induction of the responses is not limited to elongating cells in seedling roots [198]. Intriguingly, several of the responses (including gene expression, JA/SA production, lignin/callose deposition, necrosis and metabolic changes) are sensitive to osmotic manipulation, i.e., co-treatments with osmotica reduce the extent of the responses profoundly [29, 198, 199, 216]. Simultaneously, treatments with supernatants from wildtype seedlings exposed to CWD-inducing agents or with known elicitors such as OGs or flg22 do not induce phytohormone production or defence gene expression in the same manner as CWD-induced CWI impairment does [198]. These results suggest that cell wall-derived fragments are probably not primarily responsible for the effects observed after CBI treatments. Intriguingly, in *Saccharomyces cerevisiae* most, if not all, effects induced by CWI impairment are also suppressed by osmotic co-treatments, i.e., mild hyper-osmotic stress [30]. These observations suggest that turgor-sensitive processes are relevant for detection of CWI impairment. Based on the available data, different stimuli are conceivable as indicators of CWI impairment and consequently different sensors could be involved. Recently, a biophysical model has been developed, which provides a conceptual framework for investigating the responsible mechanisms [217]. One possibility is a change in the surface tension of cell walls, leading to conformational changes in CWI sensors and activation of signalling cascades. Similar mechanisms have been described in yeast and animal cells involving mechanosensitive proteins [218, 219]. Other possibilities are that CWI impairment affects organisation of the cytoskeleton, allows displacement of plasma membrane versus cell wall or causes distortion of the plasma membrane itself, events that may serve as indirect signals. These possibilities could be plant specific or similar to mechanisms contributing to detection of CWI impairment in yeast [30, 220]. The first one could involve plasma membrane localized proteins connecting the cytoskeleton with the cell wall, the second consists of the regular turgor-monitoring processes while the third one involves a plasma membrane-localized, stretch-activated calcium channel complex like MID1 CCH1 in yeast. Interestingly, the Arabidopsis-derived plasma membrane-localised calcium channels MID1-COMPLEMENTING ACTIVITY1 (MCA1) and MCA2 are capable of partially complementing MID1 CCH1 deficient yeast strains (Table 2) [221, 222]. MCA1 is required for the response to CWI impairment in Arabidopsis while both MCA1 and 2 have been implicated in mechano- and hypo-osmotic stress-perception [198, 222]. MECHANOSENSITIVE CHANNEL OF SMALL CONDUCTANCE-LIKE 2 (MSL2) and MSL3, chloroplast-localized ion channels also required for hypo-osmotic stress perception have also been implicated in CWI maintenance as well while ARABIDOPSIS HISTIDINE KINASES 1–4, required for perception of hyper-osmotic stress, seem not necessary for detection of CWI impairment [198, 223, 224]. This observation reinforces the notion that shrinking of the plasma membrane activates signalling processes distinctly different from those activated by plasma membrane expansion. Importantly, sudden plasma membrane expansion does not occur in plant cells under non-stress conditions due to the rigid cell walls.

Results from genetic analyses suggest that MCA1, the RLKs FEI2 and MALE DISCOVERER1-INTERACTING RLK 2 (MIK2) act downstream of THESEUS1 (THE1) in initiating CWD responses [198, 225]. THE1 belongs to the CrRLK1L (*Catharanthus roseus* RLK1-like) family of receptor kinases with 18 members, which are characterized by a conserved structure consisting of extracellular malectin-like domains, a transmembrane region and a cytoplasmic kinase domain [226, 227]. Homologs of THE1 have been found throughout the plant kingdom including “ancient” plants like *Marchantia polymorpha* indicating that THE1-mediated processes are widespread [226, 228]. Increasing or decreasing THE1 activity using loss- or gain-of-function alleles results in corresponding changes in CWD responses induced by CBI treatment or cell wall-degrading enzymes, implicating THE1 in CWI maintenance [198, 229]. In animals, malectin domains bind to components of the extracellular matrix [226]. For another CrRLK1L family member, FERONIA (FER), the available data suggest that FER can bind pectic polysaccharides through its malectin-like domain in vitro [153, 230]. However, the situation seems to differ in vivo since there FER binding to the cell wall apparently occurs via LEUCINE-RICH REPEAT EXTENSIN (LRX) proteins [231]. THE1, FER and several LRX proteins bind different RAPID ALKALIZATION FACTORs (RALFs) in a pH dependent manner [232–235] RALFs are small peptides, which induce changes in apoplastic pH and might modulate binding preferences of CrRLK1L proteins [233]. Recently emerged evidence suggests that the pH in the apoplast is changing dynamically in response to pathogen infection and could contribute to initiating stress-specific defence responses [236]. In contrast to THE1, loss of FER activity has profound effects on a large number of processes including biotic/abiotic stress responses, gametophytic development, mechano-perception, Ca^2+^-signalling and carbohydrate metabolism [153, 226, 237]. Based on these observations and results from biochemical studies it was proposed that FER might act as scaffold, which regulates interactions/integrates different CWI-related signals to control and coordinate downstream responses [232]. While the exact mode of action of FER remains to be determined, under standardized conditions FER and THE1 have opposite effects on CWD-induced responses like JA production and lignin deposition [198]. This implies that FER and THE1 have different functions during CWI maintenance, which is further supported by results from the functional characterization of FER indicating that FER is required during recovery from CWI impairment [153]. The authors have proposed that FER is required for confirmation that CWI has been re-established and growth can commence again. Intriguingly FER has recently been shown to regulate organization of the vacuole, reinforcing the notion that changes in the state of the cell wall might be coordinated with changes in turgor levels via FER and vacuole organization [231].

While FER and THE1 are currently the CrRLK1Ls with the strongest connections to CWI maintenance, it remains to be determined if other CWI sensors exist. The involvement of several other CrRLK1L family members in CWI maintenance has been investigated under the same conditions as FER and THE1 [198]. HERCULES1 and HERCULES2 loss of function alleles have similar but less pronounced effects than a FER knock down allele, while loss of ERULUS activity results in no significant differences to wildtype controls [198]. These results suggest that different family members may have evolved specialized functions. However, none of the CrRLK1Ls examined (including THE1 loss of function seedlings) exhibited complete loss of phytohormone production and lignin deposition in response to CBI-induced CWD [198]. This suggests that either redundancy occurs within the family or that other CWI sensors exist. A functional analysis of genes identified through a combination of transcriptomics data and FTIR-based cell wall analysis has implicated several new molecular components in the CWI maintenance [238]. Loss of the WALL STRESS RESPONSE 4 (WSR4) RLK affected cellulose levels and ISX-induced JA and lignin accumulation, indicating a role of WSR4 in CWI maintenance. In parallel, loss of the dirigent-like protein WSR1 caused mild alterations in cell wall structure, impaired ISX-induced SA accumulation and increased susceptibility to the necrotrophic fungus *Plectosphaerella cucumerina* [238].

### CWI during plant development

**Fig. 2:**
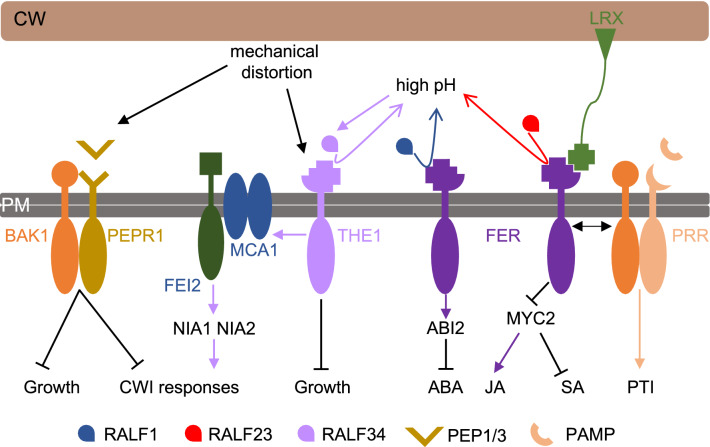
Overview of CWI signaling components, their interactions and coordination between CWI and PTI signaling. Mechanical distortion of the CW induces CWI responses through THE1 and leads to production of elicitor peptides PEP1 and PEP3, which can suppress CWI responses and growth via PEPR1/2 (represented here by PEPR1) [198]. In parallel to mechanical distortion, the action of THE1 can be regulated by RALF34, which binds to THE1 at high apoplastic pH, leading to further alkalinisation of the apoplast [233]. THE1 activates CWI responses via MCA1 and FEI2 and NIA1 NIA2-dependent processes, which repress growth actively [198]. RALF1 and RALF23 induce alkalinization of the apoplast in a FER-dependent manner [247, 250]. Both of them also affect hormone signaling pathways through FER: RALF23 by inhibiting the de-stabilizing effect of FER on the transcription factor MYC2, a master regulator of JA signaling; RALF1 by activating ABI2, a repressor of ABA signaling [247–249]. In addition, RALF23 has been shown to inhibit FER’s scaffold activity for pattern-recognition receptors (PRR) and their co-receptor BAK1, thus reducing sensitivity of the plant to respective PAMPs [232]. In addition to being involved in chemical signaling through PAMPs, RALFs and phytohormones, FER is capable of sensing physical signals from the CW, possibly through LRX proteins linking FER to CW [234]. Brown bars represents the cell wall (CW), grey bars represent the plasma membrane (PM). The white space in between the CW and PM represents the CW–PM interphase, where solutes can diffuse freely and changes in the mechanical forces are being sensed. Abbreviations are explained in main text

Contributions of CWI to plant development has been reviewed competently recently. Therefore, we will discuss here only a particular aspect [211, 226]. While ploidy levels affect cell wall formation, knowledge regarding the influence of the cell wall on plant cell cycle activity is very limited [42, 239]. Recently the impact of CBI treatments on cell cycle progression was assessed in Arabidopsis to determine if the state of the cell wall is coordinated with cell cycle activity [42]. Activity was inhibited within hours after start of treatment, while osmotic co-treatments reduced the inhibitor effects on expression of the cytokinin-regulated cell cycle regulator *CYCD3;1* and CBI-induced changes in cytokinin metabolism. Interestingly, none of the genes previously implicated in the CBI-induced responses (including THE1) are required for the changes observed in cell cycle gene expression. Instead NIA1 NIA2-dependent processes seem to be essentially required for the effects on the cell cycle. NIA1 NIA2 encode nitrate reductases, which are required for nitrogen metabolism and their mutant alleles are frequently used as genetic tools to reduce NO production [240]. Interestingly, CBI-induced phytohormone production and lignin deposition, classic hallmark responses of CWI impairment, are completely absent in *nia1 nia2* seedlings. Results from a genetic analysis place *NIA1 NIA2* downstream from *THE1* [42]. The observations also imply that additional, unknown CWI sensors exist, which detect CWI impairment arising during growth, development and interaction with the environment. Calcium-based signal transduction seems to be involved in CWI signalling, and the signals generated seem to feed into NIA1 NIA2-mediated processes, which lead to activation of downstream responses including lignin deposition and phytohormone production through additional to be determined molecular components (Fig. 2).

### A model for the plant CWI maintenance mechanism

Signals generated by the CWI maintenance mechanism have to be coordinated with signals deriving from other processes to ensure that plants respond to changes in their environment in an integrated and adaptive manner. This can also involve integration of signals generated in response to chemical and physical stimuli. While our knowledge on this topic is very limited, we will use here the process responsible for integrating signals derived from CWI maintenance and PTI as example since it provides food for thought how signal integration may work (Fig. 2). PTI is responsible for activation and modulation of defence responses in case a plant gets infected by a pathogen [241, 242]. This process involves chemical signals in the form of pathogen-associated molecular patterns (PAMPs) or damage-associated molecular patterns (DAMPs) derived from the plant itself [150]. Examples for DAMPs are cell wall fragments like xyloglucan or cellobiose, extracellular ATP or small peptides like PEP1, which can all induce defence responses in unharmed cells [103, 243, 244]. PEP1 acts in PTI as enhancer of immune responses and is activated by the calcium-regulated METACASPASE4 in response to cell integrity impairment [245, 246]. Intriguingly CWI signalling is inhibited by PEP1 and impaired PTI signalling leads to enhanced CWI signalling [198]. The relevance of CWI signalling in pathogen response is indicated by loss of THE1 or FER affecting resistance to *Botrytis cinerea* and *Fusarium oxysporum* [225, 232]. These results suggest that CWI and PTI signalling are coordinated during pathogen infection and CWI monitoring could function as backup system to induce stress responses in case PTI is impaired. In fact, it has been proposed that FER might function as scaffold protein at least in two different complexes active during PTI. This creates the possibility that FER forms the key element integrating CWI and PTI signalling [232]. In one complex, the interaction between FER and BAK1, a key regulator of PTI, modulates activation of PTI controlled responses. FER seems to be released from this complex through interaction with RALF23. This interaction controls simultaneously FER-dependent regulation of MYC2, which is responsible for control of JA and SA production [247]. In parallel, FER interaction with RALF1 regulates ABA INSENSITIVE2 (ABI2) activity, a key regulator of ABA-based signaling processes [248, 249]. Last but not least RALF1-mediated, FER-dependent inhibition of the PM-localized H^+^-ATPase PLASMA MEMBRANE PROTON ATPASE 2 (AHA2), could lead to changes in apoplastic pH [250]. Changes in apoplastic pH in turn can affect other signaling processes exemplified by the pH-dependent binding of RALF34 to THE1, possibly activating CWI signaling [233]. In the second complex FER seems to interact with LRX proteins and RALF22/23 to regulate responses to salt stress and reorganization of the vacuole [231, 234]. These results suggest that FER has a key function in integrating inputs from multiple cell wall-plasma membrane associated signalling processes and regulating downstream responses represented here by different phytohormones. More importantly this possibility would explain why FER in Arabidopsis and FER homologs in other species are involved in such a large number of biological processes [251–254].

## Concluding remarks

To summarize it has become obvious that plant cell walls in different plant species are extremely plastic. They modify their composition and structure in a highly adaptive manner and often the phenotypes (or the lack thereof) observed cannot be explained by simple functional redundancy between family members. These observations have led to proposals that in plants, like in other organisms such as yeast, a CWI maintenance mechanism exists. Recent years have seen a dramatic increase in our knowledge regarding the mode of action of the plant CWI maintenance mechanism in Arabidopsis, including the identification of several key molecular components involved. In parallel it has become obvious that homologs of these components exist in other plant species, implying that the mechanism exists there as well. This is further supported by mutant phenotypes observed, which cannot be easily explained using arguments such as genetic redundancy. While we still struggle to understand the molecular mechanisms underlying CWI maintenance, the speed of discoveries on the topic in recent years strongly suggests we head towards a thorough understanding of its mode of action in model organisms like Arabidopsis. This will enable us to apply the knowledge generated to improve the performance of food and bioenergy crops in the future in a knowledge-based manner to facilitate adaptation to a rapidly changing environment.
